# Intravascular Leiomyosarcoma in the Left Renal Vein

**DOI:** 10.5334/jbsr.1911

**Published:** 2019-10-04

**Authors:** Jae Young Lee, Seung Soo Kim

**Affiliations:** 1 Department of Radiology, Soonchunhyang University College of Medicine, Cheonan Hospital, Cheonan-si, KR

**Keywords:** Leiomyosarcoma, Renal veins, Computed tomography, Positron emission tomography computed tomography, Magnetic resonance imaging

## Abstract

**Main teaching point:** The typical imaging feature of intravascular leiomyosarcoma is a heterogeneous enhancing mass within an expanded vessel.

## Case History

**Figure 1:**
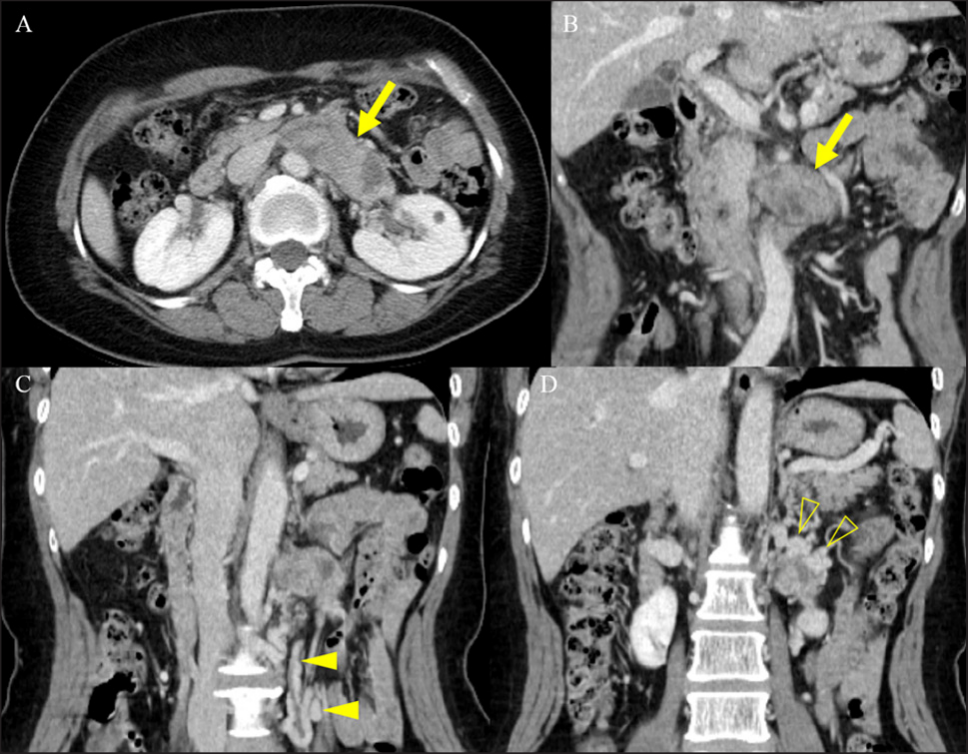


**Figure 2:**
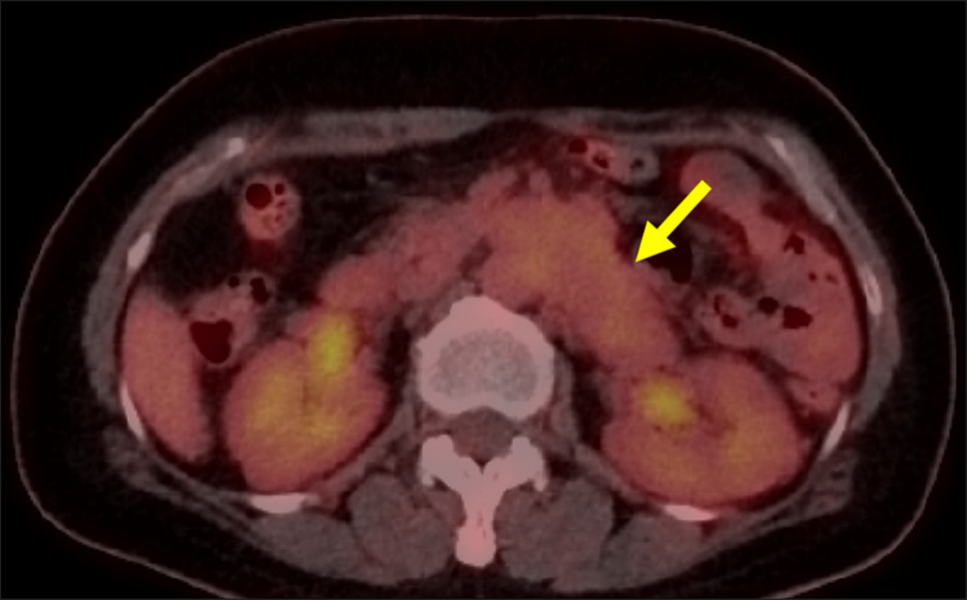


**Figure 3:**
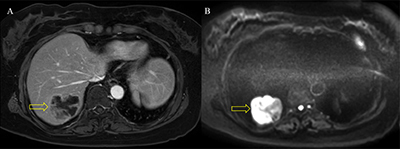


A 52-year-old woman presented with a two-month history of intermittent left flank pain. Her past medical history was unremarkable except for hysterectomy for uterine leiomyoma. Results of urinalysis and tumor markers were within the normal ranges as follows: 1–4 leukocytes and <1 erythrocytes per high-power field; carbohydrate antigen (CA 19–9), 4.52 U/ml; and carcinoembryonic antigen (CEA), 0.92 ng/ml. Contrast-enhanced computed tomography (CT) was performed for evaluation of flank pain. An axial and coronal reformatted contrast-enhanced CT image (Figure 1) showed a 7-cm, tubular, heterogeneous, enhancing mass (arrow) with a well-defined margin within the left renal vein. An elongated left ovarian vein (arrowheads) with collateral vessels (open arrowheads) was also detected. Positron emission tomography-CT image (Figure 2) revealed mild fluorodeoxyglucose uptake (max Standardized Uptake Value, 3.0) in the mass (arrow). The patient underwent mass excision and left nephrectomy and was diagnosed with leiomyosarcoma of the left renal vein. On gadoxetic acid-enhanced magnetic resonance imaging performed 19 months later (Figure 3), a recurrent metastatic lesion (open arrow) was detected in the liver, and she underwent right hemihepatectomy.

## Comment

Leiomyosarcoma is a common primary retroperitoneal sarcoma and is the most common sarcoma that arises from the blood vessels. It is classified by growth pattern into extravascular (62%), intravascular (5%), and both extravascular and intravascular (33%) types. The intravascular type of leiomyosarcomas frequently occurs in the inferior vena cava, and those arising from the renal vein are rare [1].

The CT finding of intravascular leiomyosarcomas reveals a heterogeneous enhancing lesion within an expanded vessel, which is uncommon for a thrombus. The intravascular leiomyosarcomas usually show a well-circumscribed margin with a mean size greater than 10 cm. Calcification is an uncommon feature for leiomyosarcoma. The near-total obstruction of the involved vessel may be accompanied by collateral vessels. Metastases are common in the intravascular type, and common metastatic sites are lung, liver, peritoneum, and pleura. En bloc resection of the tumor includes nephrectomy and is the treatment of choice for intravascular leiomyosarcoma in the renal vein [1].
